# Longitudinal Effect of Stroke on Cognition: A Systematic Review

**DOI:** 10.1161/JAHA.117.006443

**Published:** 2018-01-15

**Authors:** Eugene YH Tang, Obreniokibo Amiesimaka, Stephanie L Harrison, Emma Green, Christopher Price, Louise Robinson, Mario Siervo, Blossom CM Stephan

**Affiliations:** ^1^ Institute of Health and Society Newcastle University Institute of Ageing Newcastle University Newcastle upon Tyne UK; ^2^ Newcastle University Institute of Ageing Newcastle University Newcastle upon Tyne UK; ^3^ Institute of Neuroscience Stroke Research Group Newcastle University Newcastle upon Tyne UK; ^4^ Institute of Cellular Medicine Human Nutrition Research Centre Newcastle University Newcastle upon Tyne UK; ^5^ Department of Rehabilitation, Aged and Extended Care Repatriation General Hospital Flinders University Daw Park South Australia; ^6^ Department of Public Health and Primary Care Cambridge Institute of Public Health University of Cambridge Cambridge UK

**Keywords:** cognition, cognitive impairment, dementia, risk factors/global assessment, stroke, Cognitive Impairment, Risk Factors

## Abstract

**Background:**

Stroke is associated with an increased risk of dementia; however, the impact of stroke on cognition has been found to be variable, such that stroke survivors can show decline, remain stable, or revert to baseline cognitive functioning. Knowing the natural history of cognitive impairment after stroke is important for intervention. The aim of this systematic review is to investigate the longitudinal course of cognitive function in stroke survivors.

**Methods and Results:**

Three electronic databases (Medline, Embase, PsycINFO) were searched using OvidSP from inception to July 15, 2016. Longitudinal studies with ≥2 time points of cognitive assessment after stroke were included. In total, 5952 articles were retrieved and 14 were included. There was a trend toward significant deterioration in cognitive test scores in stroke survivors (8 studies). Cognitive stability (3 studies) and improvement (3 studies) were also demonstrated, although follow‐up time tended to be shorter in these studies. Variables associated with impairment included age, ethnicity, premorbid cognitive performance, depression, stroke location, and history of previous stroke. Associations with APOE*E4 (apolipoprotein E with the E4 allele) allele status and sex were mixed.

**Conclusions:**

Stroke is associated with an increased risk of cognitive decline, but cognitive decline is not a consequence. Factors associated with decline, such as sociodemographic status, health‐related comorbidity, stroke history, and clinical features could be used in models to predict future risk of dementia after stroke. A risk model approach could identify patients at greatest risk for timely intervention to reduce the frequency or delay the onset of poststroke cognitive impairment and dementia.

**Clinical Perspective**

**What Is New?**

- Cognitive outcome following a stroke is dependent on sociodemographic, health, and stroke‐related risk factors and the timing of cognitive assessment.

**What Are the Clinical Implications?**

- Poststroke patients need to have their cognitive function followed up over time to ensure that cognitive decline is noted early.
- Known risk factors associated with poststroke cognitive decline could be incorporated into risk scores to ensure timely detection of poststroke cognitive decline.

## Introduction

Stroke is the second most common cause of acquired cognitive impairment, which predisposes patients toward institutionalization, disability, increased mortality, and poorer quality of life.^1^, ^2^, ^3^ With an aging population and a decline in mortality after stroke,^4^ the rates of poststroke cognitive impairment will increase. Despite being as common as other neurological deficits, such as motor and sensory, cognitive impairment is often overlooked in the follow‐up of stroke survivors unless they have progressed to dementia.^5^ This may well be because these patients are able to maintain some level of personal independence despite poor cognitive recovery.^6^

It has been found that stroke survivors may show no cognitive deficits or may decline, initially decline and then improve, remain stable, or progress to dementia over time.^7^, ^8^ Mixed findings may be related to differences in the cognitive tests used and test timing, history of previous stroke, stroke location, large‐ and small‐vessel disease, population sample (clinical versus population based), ethnicity, and the presence of neurodegenerative pathology.^9^ Nevertheless, it is also possible that the initial poststroke cognitive state may reflect prestroke cognitive decline^10^ or delirium.^11^ There is a drive toward detecting long‐term cognitive outcomes after stroke to explore prevention; however, a preferred testing strategy is lacking, making cross‐study comparison difficult.^12^

The aim of this systematic review was to assess the longitudinal pattern of cognitive function in stroke survivors and to determine those factors associated with change over time. Recognizing the natural history of cognitive impairment after stroke is vital for informing early treatment and preventative strategies.

## Methods

The data, analytic methods, and study materials will be made available to other researchers for purposes of reproducing the results or replicating the procedure. This material can be made available by the corresponding author on reasonable request.

### Search Strategy and Selection Criteria

This systematic review was undertaken in accordance with the PRISMA (Preferred Reporting Items for Systematic Reviews and Meta‐Analyses) statement.^13^ The review was registered with PROSPERO (CRD42014015018). Three electronic databases—Medline, Embase, and PsycINFO—were searched using OvidSP from inception to July 15, 2016; searches were restricted to human studies and articles published in English. Predefined and Boolean search terms were used, including stroke, (cognit* or neuropsych*), and (progress* or longitudinal or decline or prospective). Longitudinal studies with ≥2 time points of cognitive assessment after stroke were included. No distinction was made regarding the sampling framework (clinic, hospital, or population based), the number of strokes, or the timing of cognitive assessments after stroke or cognitive battery used. Studies in which baseline and subsequent incident stroke cases found at follow‐up were analyzed together were included. No distinction was made regarding the mechanism of stroke, and studies were not excluded if stroke was not confirmed using neuroimaging. All participants were adults who were aged ≥50 years and free from dementia. Randomized controlled trials and cognitive rehabilitation studies were excluded. Studies in which the only outcome was a diagnosis of dementia were excluded because this was the subject of a previous review.^14^ Studies were excluded if change in cognitive function over time in the stroke group was not reported (ie, studies that only compared cognitive outcomes in stroke patients versus controls were excluded). Studies were also excluded if they reported percentages of decline rather than actual test scores or did not report statistical comparison of change in cognitive performance over time.

Four authors (O.A., E.Y.H.T., E.G., and S.L.H.) independently searched the article titles and abstracts. If the article could not be rejected with certainty based on title or abstract alone, then the full text of the article was obtained. Discrepancies between authors were resolved by consensus, and if this was not possible, then they were resolved by a third author (B.C.M.S.). Four authors (O.A., E.Y.H.T., E.G., and S.L.H.) carried out evaluation of full‐text articles. Consensus or a third author resolved disagreements. The reference lists of the full‐text articles and any relevant reviews were searched for potential eligible references.

### Data Extraction

Data extracted included study design, sample size, demographic characteristics, inclusion or exclusion criteria, definition of stroke, cognitive test battery, and results. Three authors (E.Y.H.T., S.L.H., and E.G.) extracted data independently, and any discrepancies were resolved through consensus or discussion with a third author (B.C.M.S.). Because of the heterogeneity in the study design (eg, variation in follow‐up time, cognitive test battery used), a meta‐analysis was not possible.

## Results

Figure shows the results of the electronic search and article‐selection process. The search identified 9365 articles, of which 3413 were duplicates and thus removed. After reviewing titles and abstracts, 238 articles were retained for full‐text review. The main reasons for exclusion were that the study population age was <50 years, cognitive measures were not reported, and a cross‐sectional design was used. Fourteen articles met the inclusion criteria.

**Figure 1:**
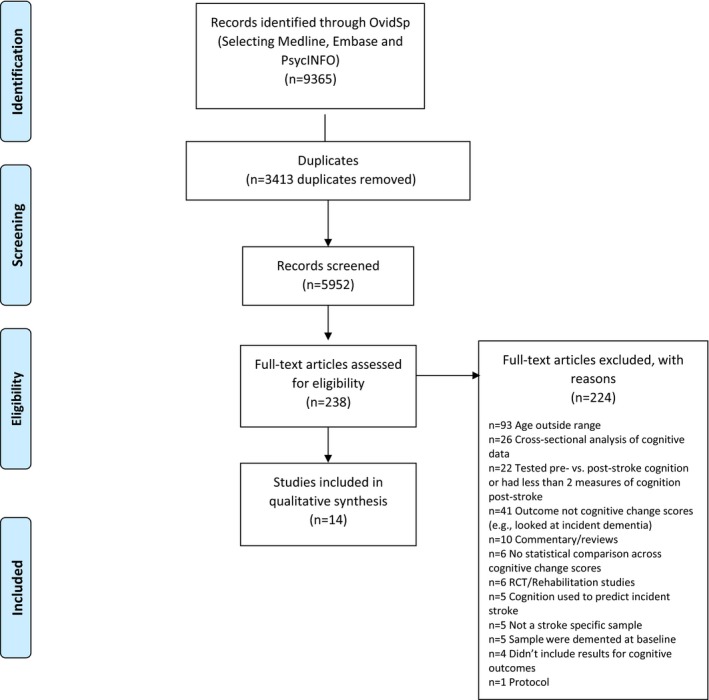
Flow diagram showing article selection. RCT indicates randomized controlled trial.

### Study Characteristics

Characteristics of the included studies and detailed cognitive outcomes are shown in Tables S1 and S2, respectively. The number of participants at baseline ranged from 50^15^ to 1187.^16^ Follow‐up ranged from 3 weeks^17^ to 6 years.^15^, ^18^, ^19^ The cohorts included stroke‐specific populations^17^, ^20^, ^21^, ^22^, ^23^ and population‐based studies.^15^, ^16^, ^18^, ^19^, ^24^, ^25^, ^26^, ^27^, ^28^ The majority of studies were conducted in the United States (n=4),^16^, ^18^, ^25^, ^26^ followed by the United Kingdom (n=2),^20^, ^27^ Israel (n=2),^22^, ^23^ the Netherlands (n=2),^15^, ^24^ Germany (n=1),^19^ India (n=1),^28^ Norway (n=1),^21^ and Japan (n=1).^17^ Stroke case ascertainment included hospital‐based diagnosis,^20^, ^21^, ^22^, ^23^, ^27^ self‐report,^15^, ^25^ general practitioner records,^19^ self‐report confirmed through medical records and/or expert review,^16^, ^18^, ^24^, ^26^, ^28^ and not specified.^17^ Only 2 studies used magnetic resonance imaging data.^22^, ^23^

### Cognitive Assessments

The Mini Mental State Examination (MMSE; total,^16^, ^17^, ^21^, ^24^, ^28^ subtests scores,^26^ and modified MMSE [3MSE]^25^) was the most commonly used cognitive assessment. Other batteries for assessing global cognitive function included the Montreal Cognitive Assessment (MoCA),^22^, ^23^ the Cambridge Cognitive Assessment (CAMCOG),^27^ the revised CAMCOG (CAMCOG‐R),^20^ and the Repeatable Battery for the Assessment of Neuropsychological Status (RBANS).^21^ Domain‐specific cognitive tests included the Auditory Verbal Learning Test (immediate and delayed recalled),^24^ the Alphabet Coding Task,^15^, ^24^ East Boston Test,^16^ the Symbol Digits Modalities Test,^16^ subtests of the neuropsychological test battery of the Consortium to Establish a Registry for Alzheimer's Disease,^19^ 2 subsets of Raven's Coloured Progressive Matrices,^15^ and the word‐list delayed recall of the Spanish and English Verbal Learning Test.^25^ The computerized neuropsychological assessment NeuroTrax was used by 2 studies but in the same cohort.^22^, ^23^

### Study Sampling Framework

The majority of cohorts were population based (n=8),^15^, ^16^, ^18^, ^19^, ^24^, ^25^, ^26^, ^28^ although 5 studies were hospital based,^17^, ^20^, ^21^, ^22^, ^23^ and the sampling framework was unclear in 1 study.^27^ The studies demonstrating cognitive decline tended to be population‐based cohorts with longer follow‐up (3 years^28^ to 6 years^15^, ^18^). Studies demonstrating cognitive recovery were all hospital‐based cohorts with shorter follow‐up (3 weeks^17^ to 13 months^21^). Cognitive outcomes were also reported in separate studies from the same population in 2 cohorts: a hospital‐based cohort (Tel Aviv Brain Acute Stroke Cohort)^22^, ^23^ and a population‐based cohort (Longitudinal Aging Study Amsterdam).^15^, ^24^

### Cognitive Function After Stroke

The impact of stroke on cognitive function over time was mixed as shown in the Table.

**Table 1:** Characteristics and Cognitive Findings From Included Studies (n=14)

Author	N	Sampling Framework	Follow‐up	Cognitive Assessment	Key Findings	Risk Variables
Decline						
Comijs, 2009^15^	50 T1, 90 T2, 84 T3	Population‐based cohort	Maximum 6 y	MMSE, RCPM, ACT, AVLT (immediate and delayed recall)	Significant decline in memory (immediate and delayed recall) and information processing speed No significant change in global cognitive function	N/A
Rajan, 2014^16^	1187	Population‐based cohort	Mean 4.2 y (SD 3.9)	East Boston Test (immediate and delayed story recall), Symbol Digits Modalities Test, MMSE (total and orientation scores)	Significant decline in global cognitive function	Increased risk of decline among black patients compared with white patients (all tests)
Ghosal, 2014^28^	283	Population‐based cohort	Maximum 3 y	MMSE (Bengali version)	Significant decline in global cognitive function	Global impairment more common in women, higher age of onset of stroke, and people with higher depression scores
Levine, 2013^25^	151	Population‐based cohort	Mean poststroke follow‐up of 3.6 y for women and 3.4 y for men	Modified MMSE (3MSE), Word‐List Delayed Recall of the Spanish and English Verbal Learning Test (SEVLT)	No significant change in global cognitive function or verbal memory No significant overall sex differences	No effect of systolic blood pressure on global cognition
Reitz, 2006^26^	97	Population‐based cohort	Maximum 5 y	Orientation (MMSE items), Boston Naming Test, Controlled Word Association Test, category naming, Boston Diagnostic Aphasia Evaluation (Complex Ideational Material and Phrase Repetition), WAIS‐R similarities subtest, nonverbal Identities and Oddities subtest of the Mattis Dementia Rating Scale, Rosen Drawing Test, Benton (matching), Benton Visual Retention Test and the Selective Reminding Test	Significant decline in memory No significant change in abstract/visuospatial or language	Significant decline in memory in men and abstract/visuospatial in APOE*E4‐negative patients
Ben Assayag, 2015^22^	298	Hospital‐based cohort	Maximum 2 y	MoCA and computerized global cognitive score (including memory, executive functions, visuospatial perception, verbal function, attention and motor skills)	Significant decline in global cognition in those taking longer to complete the TUG	Multivariable model: Age ≥75 y, TUG score >12 s at 6 mo after stroke, MoCA score 6 mo after stroke
Tene, 2016^23^	306	Hospital‐based cohort	Maximum 2 y	As above	Significant decline in global cognition, memory, executive functioning and visuospatial in those with higher admission and six‐month GDS scores; attention also declined in those who had higher GDS scores at 6 months	Multivariable model: MoCA score at hospital admission, age ≥75 y, GDS score ≥6 (admission and 6 mo after stroke)
Toole, 2004^18^	5364	Population‐based cohort	Maximum 6 y	3MS	Significant decline in global cognitive function	Left‐hemisphere (highest decline) and right‐hemisphere strokes
Stability						
Kohler, 2012^19^	3214	Population‐based cohort	Maximum 4.5 y	CERAD verbal fluency and recall (immediate and delayed) tasks	No significant change in verbal fluency, immediate or delayed recall	Not reported
Rowan, 2007^27^	126	Unclear	Maximum 27 months	CAMCOG and the Cognitive Drug Research computerized battery	N/A	No significant decline in global cognitive function when stratified by homocysteine levels
Dik, 2000^24^	53	Population‐based cohort	Mean 3.1 y (SD 0.2)	MMSE, AVLT (immediate and delayed), Coding Task (information processing speed)	No significant change in global cognition, memory, or information processing speed (adjusted models)	Lowered risk for global cognitive decline for APOE*E4 carriers (not significant)
Recovery						
Leeds, 2001^20^	83	Hospital‐based cohort	Maximum 3 months	CAMCOG‐R, Weigl color form sorting test, Raven's matrices	Significant improvement in global and executive function	Depression influenced executive function and CAMCOG‐R scores
Wagle, 2010^21^	104	Hospital‐based cohort	Mean 408.4 d (SD 41.2)	RBANS and MMSE	Significant improvement in visuospatial/constructional, delayed memory and global cognition (RBANS only) No significant change in global cognition (MMSE), immediate memory, language and attention	Multivariable model: Presence of APOE*E4, prestroke cognitive reduction, previous stroke, and neurological impairment
Suzuki, 2013^17^	57	Hospital‐based cohort	Maximum 3 wk	MMSE	Significant improvement in global cognitive function	Not reported

ACT indicates Alphabet Coding Task; AVLT, Auditory Verbal Learning Test; CAMCOG, Cambridge Cognitive Assessment; CAMCOG‐R, Cambridge Cognitive Assessment (Revised); CERAD, Consortium to Establish a Register for Alzheimer's Disease; GDS, Geriatric Depression Score; MMSE, Mini Mental State Examination; MoCA, Montreal Cognitive Assessment; N/A, not assessed; RBANS, Repeatable Battery for the Assessment of Neuropsychological Status; RCPM, Raven's Colored Progressive Matrices; SEVLT, Spanish and English Verbal Learning Test; T, time point; 3MSE, Modified Mini Mental State Examination; TUG, Timed Up and Go; WAIS‐R, Wechsler Adult Intelligence Scale–Revised.

#### Global cognitive function

Most studies (n=12) included a measure of global cognitive function. Of these, 3 studies^16^, ^18^, ^28^ reported significant decline (follow‐up: 3–6 years), 3 studies^15^, ^21^, ^25^ reported no change (follow‐up: 13 months to 6 years), and 3 studies^17^, ^20^, ^21^ reported significant improvement (follow‐up: 3 weeks to 13 months) over time. In stratified analyses (4 studies), it was found (1) that although there was no significant decline in global function (3MSE score), over 3 years of follow‐up in the whole sample with sex‐stratified analysis, both men and women showed significant changes in 3MSE errors (worse in men than women, but no significant sex differences)^25^; (2) that stroke patients with slower physical performance, measured using the Timed Up and Go test, performed significantly worse on a computerized global cognitive test battery compared with stroke patients with faster physical performance (follow‐up duration: 2 years)^22^; (3) that stroke patients with comorbid depression performed significantly worse on global cognitive scores compared with stroke patients without depression (follow‐up duration: 2 years)^23^; and (4) that there was no significant difference in CAMCOG scores when stroke patients were stratified by homocysteine levels (follow‐up duration: 27 months).^27^

#### Memory

Six studies included tests of memory.^15^, ^19^, ^21^, ^24^, ^25^, ^26^ Of these, 2 reported significant decline including impairments in immediate and delayed recall (follow‐up: 6 years)^15^ and visual memory (follow‐up: 5 years).^29^ Four studies reported no significant change in measures of verbal memory (follow‐up: >3 years),^25^ immediate memory (follow‐up: 13 months),^21^ or immediate and delayed recall (follow‐up: 3.1–4.5 years).^19^, ^24^ One study found an improvement in delayed memory over 13 months of follow‐up.^21^ In stratified analyses, 1 study reported significant decline in memory for those with higher Geriatric Depression Scale (GDS) scores.^23^ One study reported a significant decline in memory over 5‐year follow‐up and was also found to be strongest for men compared with women.^26^

#### Nonmemory

Five studies included nonmemory tests.^15^, ^19^, ^21^, ^24^, ^26^ One study reported a significant decline in information processing speed over 6 years of follow‐up.^15^ Three studies reported no changes in nonmemory performance including measures of abstract reasoning,^26^ visuospatial ability,^26^ verbal fluency,^19^ attention,^21^ information processing speed,^24^ and language performance^21^, ^26^ (follow‐up: 13 months to 5 years). One study reported significant improvement in executive function over 3 months follow‐up,^20^ and another reported significant improvements in visuospatial/constructional performance over 13 months follow‐up.^21^ In stratified analysis, 1 study reported significant declines in executive function and visuospatial domains for those with higher admission and 6 month GDS scores; attention also declined in those who had higher GDS scores at 6 months.^23^ A further study reported a significant decline in abstract/visuospatial scores in patients who were negative versus positive for APOE*E4 (apolipoprotein E with the E4 allele).^26^

### Risk Factors for Poststroke Cognitive Decline

Risk factors for cognitive impairment included ethnicity (greater risk in black patients compared with white patients),^16^ depression,^23^, ^28^ increased age,^22^, ^23^, ^28^ sex (mixed results^25^, ^28^), APOE*E4 status (mixed findings^21^, ^24^, ^26^), poorer cognitive performance after stroke,^22^ stroke location (left and right hemisphere),^18^ and a previous history of stroke.^21^ Findings for sex were mixed: 1 study found no sex differences,^25^ 1 found a greater risk of global impairment in women,^28^ and another found a greater risk of decline in memory in men.^26^ In 1 study, systolic blood pressure was not associated with global cognitive function >3 years after stroke in either men or women.^25^

## Discussion

In this systematic review, the effect of stroke on longitudinal changes in cognitive function before a diagnosis of dementia was found to be mixed depending on the cognitive domain tested and methodology factors (eg, follow‐up time). Furthermore, risk factors traditionally associated with cognitive decline, including APOE*E4 status^24^, ^26^ and systolic blood pressure level,^25^ did not have the same expected effect in stroke‐specific samples. Accurate identification of stroke survivors at highest risk of cognitive decline is important and could be used to identify people for early intervention and participation in clinical trials.

Regarding global cognitive function, the majority of studies reported decline,^16^, ^18^, ^22^, ^23^, ^25^, ^28^ whereas 5 reported no change.^15^, ^21^, ^24^, ^25^, ^27^ In contrast, 3 studies utilizing different cognitive batteries (MMSE^17^, CAMCOG‐R,^20^ and RBANS^21^) reported recovery. Recovery as measured by MMSE and CAMCOG‐R could be due to a combination of the small sample size of the study (57 patients^17^ and 83 patients^20^) and short follow‐up (3 weeks^17^ and up to 3 months^20^). Furthermore, the study using the RBANS had a similarly small sample size (n=104) and short follow‐up (13 months) and restricted the study population to those fulfilling requirements for rehabilitation, meaning that the study would have excluded the more severe strokes and thus potentially those at greater risk of cognitive decline.^21^ When stroke survivors were followed up for a longer period of time (eg, ≥3 years), a significant decline in global cognition was reported.^16^, ^18^, ^28^ Given that age is the biggest risk factor for incident dementia,^30^ future studies could look at whether or not age of onset of stroke and disease duration determines the longitudinal cognitive trajectory after stroke. This would involve comparing stroke cohorts that had younger onset with an older stroke population and following their cognition over time. This approach could help describe global cognitive recovery in all stroke populations and assess who is at greatest risk of cognitive nonrecovery.

When assessing domain‐specific function the results were mixed. Studies identified improvements in executive function,^20^ visuospatial/constructional performance,^21^ and memory (delayed)^21^ over 3 to 13 months of follow‐up. When participants were followed for longer periods (eg, ≥3 years), studies reported significant decline in memory (in some studies^15^, ^26^ but not all^18^, ^23^, ^28^) and no significant change in abstract/visuospatial performance.^26^ Findings were mixed for information‐processing speed, with 1 study reporting no significant change over 3 years of follow‐up^24^ but another reporting significant decline over 6 years of follow‐up.^15^ Two studies also found no significant change in language performance.^21^, ^26^ These mixed findings could be driven by varying sample size and heterogeneity in study design as well as by differences in the length of follow‐up and medical treatments. These results highlight the importance of testing different cognitive domains in stroke survivors and the need to develop a consensus cognitive battery to allow cross‐study comparison. Further work could assess the effect of stroke severity, subtypes, or locations on cognitive domains and factors that could assist in cognitive recovery.

Across studies, risk factors for cognitive decline included demographic factors (age, sex, ethnicity), neuropsychiatric symptoms (depression), disease‐related comorbidity (previous stroke), poorer baseline cognitive tests, genetic factors (ie, APOE*E4 status), function (balance and gait), and the nature of the stroke itself (stroke location). Factors such as arterial hypertension and the number of cerebral infarcts have been shown to be prognostic variables of cognitive deterioration.^31^ However, not all factors were consistently observed to increase risk, including sex and APOE*E4 status. With regard to sex, global impairment was found to be more common in women in 1 study,^28^ which is comparable to existing literature.^32^ In contrast, in another study, although there was no significant sex difference, global cognitive decline was found to be more severe in men.^25^ However, this study was performed only in older Mexican Americans and may reflect only the relative risk found for this ethnicity. When specific domains were tested, when stratified by sex, men were found to show significant decline in memory.^26^ However, the sample size for men was much smaller than that for women (n=27 versus n=70, respectively), which raises issues of statistical power. Regarding APOE*E4 status, 1 study (n=19 who were APOE genotype 4/−) found a significant association between stroke and decline in abstract/visuospatial performance in those without the APOE*E4 allele.^26^ Another (n=27 APOE*E4 positive)^24^ found that stroke patients without the APOE*E4 allele showed faster decline on global cognition, although this was not statistically significant and a synergistic effect was not observed.^24^ In contrast, yet another study found that being an APOE*E4 carrier was predictive of cognitive impairment at 13 months after stroke, but again, the sample size was small (n=25).^21^ Given the inconsistency of these results with small sample sizes, further research is warranted to identify whether APOE*E4 carriers with a history of stroke are at a higher risk of future poststroke cognitive impairment.

A number of risk scores have been developed to predict dementia in whole populations, with many using modifiable risk factors (eg, vascular risk factors^33^, ^34^) with the hope that modifying these factors could alter cognitive trajectory. A risk model approach could be used in stroke populations, incorporating some of these variables identified in this review to predict poststroke dementia.^35^ A number of risk scores have been developed recently to predict poststroke dementia (3 months after stroke, area under the curve: 0.74)^36^ and cognitive impairment (6 months after stroke, area under the curve: 0.83).^37^ Our review, however, shows that cognitive decline seems to become more apparent over a longer follow‐up period, and thus new models could be developed to predict poststroke cognitive impairment and dementia over longer time periods. Currently there are no specific biomarkers that can help discriminate between those at risk and those with better prognosis.^38^ There is evidence, however, of a strong relationship between inflammation markers and cognitive performance,^39^ and this will need further evaluation before being used in potential risk models. Although neuroimaging variables were used in the cognitive impairment model,^37^ evidence shows that data from magnetic resonance imaging do not significantly improve prediction in all‐cause dementia models.^40^ Similarly, there may be less focus on incorporating vascular risk factors into these models because results from a recent clinical trial found that intensively managing vascular risk factors in stroke survivors did not alter cognition after 2 years.^41^

### Strengths and Limitations

This study has a number of strengths. We performed a systematic search of all studies focusing on older aged samples. Furthermore, we did not restrict our search by cognitive domain. This is important in stroke samples, for which overall cognitive improvement may be explained by significant improvements in some nonmemory domains but individuals may still show persisting memory deficits. We also ensured that the included studies had statistical comparisons of change in cognitive test scores over time. Nevertheless, there are limitations. Only studies in English were included, and the majority of studies were in white populations. Consequently, the results may not extrapolate to nonwhite samples. Studies were also excluded if the sample baseline age was ≤50 years because stroke before age 50 is uncommon, and these patients may have a different risk profile than the older population.

## Conclusions

Cognitive outcomes after stroke can be variable, and standardized assessment tools together with recommended time intervals for testing are needed. Determinants of poststroke cognitive decline are important to clarify, particularly if these patients are different from the nonstroke population; interventions may need to be tailored specifically to stroke survivors. A number of risk factors for cognitive decline, particularly in global functioning, in stroke survivors have been found, such as age, sex, stroke location, and medical comorbidities (depression), and could be incorporated into a risk tool to identify stroke survivors at highest risk of cognitive decline over short and long durations of follow‐up.

## Sources of Funding

Tang is supported by a National Institute for Health Research (NIHR) Doctoral Research Fellowship (DRF‐2015‐08‐006). Robinson is supported by an NIHR professorship (NIHR‐RP‐011‐043) and an NIHR Senior Investigator award. Siervo is supported by a Medical Research Council Grant (MR/N007921/1).

## Disclosures

None.

## Supporting information


**Table S1.** Study Characteristics
**Table S2.** Baseline and Follow‐up Cognitive Measures
