# Supplementary material for: Longitudinal Effect of Stroke on Cognition: A Systematic Review
Source: J Am Heart Assoc. 2018 Jan 15;7(2):e006443. doi: 10.1161/JAHA.117.006443 (PMC5850140; doi:10.1161/JAHA.117.006443)
Supplement: Supplementary file 1 — Table S1. Study Characteristics Table S2. Baseline and Follow‐up Cognitive Measures [file JAH3-7-e006443-s001.pdf]

SUPPLEMENTAL MATERIAL

Table S1. Study Characteristics

Author, Year	Data Resource	Demographic Data	Frequency and Timing of Cognitive Assessment	Stroke Cases and Definition Reported
Comijs 2009[1]	LASA, population based study, the Netherlands (50 T1, 90 T2, 84 T3)	Mean age (total sample)=72.1 (Range: 55-85)<br>Mean education (total sample)=9.0 years<br>Male 51.5% (total sample)	Up to six years<br>Time 1 = 1992-93, Time 2 = 1995-96, Time 3 = 1998-99	Self-reported
Rajan 2014[2]	Chicago Health and Aging Project<br><br>Baseline n=1187	Mean age=73.7 years (SD=6.3)<br>Mean (SD)<br>Mean education=12.0 years (SD=3.4)<br>Male 41%	Mean follow-up time after incident stroke was 4.2 years (SD=3.9).	Self-report ischaemic and haemorrhagic
Ghosal 2014[3]	Urban community in Kolkata<br><br>3 annual visits, baseline year (n = 283), first follow-up (n = 220), second follow-up (n = 181), third follow-up (n = 159)	Mean age (SD): 64.27 +/- 13.08 years<br>Male 51.9%<br>Female 48.9%<br>Mean education (SD): 5.42 +/- 4.84 years	Bengali versions of the Mini Mental State Examination (BMSE); Baseline and 3 annual follow-up visits	Cases initially screened by field workers using a validated WHO-based questionnaire. Screened patients were further examined by field physicians and the findings reviewed by senior neurologists.<br><br>WHO definition
Levine 2013[4]	Sacramento Area Latino Study on Aging (SALSA cohort) (1576, 151 with	Male (n=655, mean age=70.2, SD=6.7);	Yearly for ten years. Incident first-ever stroke mean years of follow-up: women 3.6 years, 3.4 for men	Participant self-report of a physician

	incident first-ever stroke during ten years of follow-up)	Female (n=921, mean age=70.<br><br>Incident first-ever stroke: 72 +/- 8 years<br>655 Males, 921 Females. Incident first-ever stroke: 66 men, 85 women<br><br>Male (n=655, mean education =8.0 years, SD=5.6); Female (n=921, mean education =6.9 years, SD=5.1); Education: First-ever stroke: male 7.6 (SD 5.6) years, female 7.0 (SD 5.9 years)		diagnosis during following up or stroke as cause of death on death report
Reitz 2006[5]	Longitudinal study of Medicare recipients in northern Manhattan (1271 (Stroke cases = 97))	Mean age = 76.2 years, SD=6.0<br>69.6% female<br>Mean education = 8.6 yrs, SD=4.6	Baseline data (1992-1994), follow up data during sequential intervals of approximately 18 months (1994-1996, 1996-1997, 1997-1999) – 5 year interval.	Participant/ informant interview; confirmed by medical records (85% included brain imaging), remainder by direct exam<br><br>WHO criteria
Suzuki 2013[6]	First round: 57, Second round 43	First round mean age 73.5 years (SD 9.3) Second round mean 72.4 (SD 10.8)<br>First round Female 56.1%<br>Second round Female 55.8%	Initial assessment from onset of stroke (baseline assessment) and then at 1 week (second set of assessments) and 2 weeks (third set of assessments) after the baseline assessment.<br><br>Second round of data collection: baseline assessment and at 1, 2, and 3 weeks after the baseline assessment in each individual.	NOT REPORTED
Ben Assayag 2015[7]	Tel Aviv Brain Acute Stroke Cohort (TOBASCO) Study (n – 298)	Mean age 66.7+/-9.6 years<br>62.4% male (n = 186)<br>Mean education 13.2 (SD 3.7) years	Baseline MoCA and NeuroTrax computerised cognitive testing and then repeated at 6, 12 and 24 months following the event. The average of the 6 index scores (memory, executive functions, visuospatial perception, verbal function, attention and motor skills) was computed as the global cognitive score.	Mild to moderate first-ever acute ischaemic stroke
Tene 2016[8]	Tel Aviv Brain Acute Stroke Cohort (TOBASCO) Study	Mean age (SD): 67.1 (10.0) years)<br>n (%) males 182 (59.5)	Baseline MoCA and NeuroTrax computerised cognitive testing and then repeated at 6, 12 and 24 months following the event. The average of the 6 index scores (memory, executive functions, visuospatial perception, verbal	Mild to moderate first-ever acute

	Baseline = 306, 6, 12 and 24 month follow-up	Mean education (SD): 13.2 (3.7) years	function, attention and motor skills) was computed as the global cognitive score.	ischaemic stroke
Toole 2004[9]	Population based cohort (Cardiovascular Health Study) (n = 5364)	≥65	Follow-up visits between 1992- 1998	Self-report and confirmed by medical record
Kohler 2012[10]	Ageing, Cognition and Dementia in Primary Care patients (AgeCoDe), Germany (3214 at baseline, 321 cases)	Mean age (total sample)=79.7 (SD=3.6, range: 75-98)<br>Education (as measured by the Comparative Analysis of Social Mobility in Industrial Nations classification):<br>Low (1988) 61.9%<br>Middle (883) 27.5%<br>High (343) 10.7%<br>Male 34.5% (total sample)	Three follow-ups, every 18 months after baseline (between 2003 – 2009)	Primary care based records - stroke or TIA
Rowan 2007[11]	N = 126	Median age=79.4 years<br>Males – n=94 (55.3%)	3, 15 and 27 months post stroke	Clinical and CT scan evidence-based diagnosis of stroke<br><br>Oxford community Stroke Project classification
Dik 2000[12]	LASA, the Netherlands<br><br>1,224 in total sample, 53 stroke patients	Age Range 65-85 years<br><br>Stroke (n=53, Mean=74.6 years, SD=6.7).<br><br>Stroke (male=37, 69.8%).<br><br>Stroke (Mean education =8.5 yrs, SD=3.4).	Mean follow-up=3.1 (SD=0.2) years	GP diagnosis of stroke. When GP diagnosis not available participant self-report (n=211)

Leeds 2001[13]	Admissions to stroke rehabilitation unit (n = 83)	60+ years (Mean age=75.4 years, SD=8.1)<br>44 male, 39 female	1 month (mean=4.14 weeks, range 1-5 weeks) and 3 months post stroke	93 stroke confirmed by CT scan; rest based on clinical history and physical examination
Wagle 2010[14]	Admissions to stroke rehabilitation unit of Ulleval University Hospital (Oslo, Norway) (n = 104)	Cognitive impairment group (n=52, age mean=81.0 SD=9.5).<br><br>No cognitive impairment group (n=52, age mean=78.0 SD=20.3)<br><br>Cognitive impairment group (n=52, females=23, 44%).<br><br>No cognitive impairment group (n=52, females=25, 47%)<br><br>Cognitive impairment group (n=52, Mean education =11.0 years SD=5.0).<br><br>No cognitive impairment group (n=52, Mean education =11.0 years SD=3.8)	12-15 months post stroke (Mean=408.4 days, SD= 41.2)	Ischemic or hemorrhagic

Abbreviations

ACT, Alphabet Coding Task; AD, Alzheimer's Disease; ADL, Activities of Daily Living; AVLT, Auditory Verbal Learning Test; BMSE, Bengali versions of the Mini Mental State Examination; CAMCOG, Cambridge Cognitive Assessment; Cambridge Cognitive Assessment ; CAMCOG-R, Cambridge Cognitive Assessment; Cambridge Cognitive Assessment (Revised); CERAD, Consortium to Establish a Register for Alzheimer's Disease; COGFAST, Cognitive Function After Stroke; CT, Computed Tomography; DSM, Diagnostic and Statistical Manual of Mental Disorders; GP, General Practitioner; LACI, Lacunar infarct; LASA, Longitudinal Aging Study Amsterdam; M, Mean; MMSE, Mini Mental State Examination; MoCA, Montreal Cognitive Assessment; MRC, Medical Research Council; NIHSS, National Institutes of Health Stroke Scale; PACI, Posterior anterior circulation infarct; POCI, posterior circulation infarct; RCPM, Raven's Colored Progressive Matrices; SD, Standard Deviation; TIA, transient ischaemic attack; WAIS-R, Wechsler Adult Intelligence Scale-Revised; WHO, World Health Organisation

Table S2 Baseline and Follow-up Cognitive Measures

Author, Year	Baseline Measures	Follow-up Measures	Grouped Results and Outcome
Comijs 2009[1]	Mean (±SD)<br><br>General Cognitive Functioning (MMSE):<br>T1 27.5±2.0<br>Fluid Intelligence<br>T1 18.0±3.9<br>Information processing speed<br>T1 24.3±6.9<br>Immediate AVLT<br>T1 7.7±2.5<br>Delayed AVLT<br>T1 5.1±2.6	Mean (±SD)<br><br>General Cognitive Functioning (MMSE):<br>T2 27.2±2.4<br>T3 27.2±2.4<br>Fluid Intelligence<br>T2 17.6±4.0<br>T3 17.2±4.0<br>Information processing speed<br>T2 23.3±7.1<br>T3 23.2±6.9<br>Immediate AVLT<br>T2 8.0±2.7<br>T3 7.7±2.7<br>Delayed AVLT<br>T2 5.7±3.0<br>T3 5.3±2.9	Interactions between time and stroke (mean differences in cognitive function) between those with and those without stroke<br><br>Information processing speed (ACT)<br>T1 -1.08, T2 -2.78, T3 -3.40 ($p < 0.05$)<br><br>Immediate Recall (AVLT)<br>T1 0.01, T2 -0.35, T3 -0.72 ($p < 0.04$)<br><br>Delayed Recall (AVLT)<br>T1 0.10, T2 -0.41, T3 -0.92 ($p < 0.005$)<br><br>MMSE β=-0.26 (-0.69/0.16); RCPM β=-0.73 (-1.32/-0.14); ACT β=-1.97 (-2.78/-1.16); AVLT (immediate) b=-0.44 (-0.83/-0.06); AVLT (delayed) β=-0.56 (-0.95/-0.17)<br><br>Compared to no-stroke, stroke had a higher rate of decline for information processing speed (p=0.05) and memory (immediate p=0.04, delayed p=0.005)
Rajan 2014[2]	MMSE Mean (SD): 26.3 (4.2)<br><br>Delayed recall Mean (SD): 7.7 (3.0)<br><br>Immediate recall Mean (SD): 8.3 (2.6)<br><br>Symbols digit Mean (SD): 28.4 (13.7)<br><br>Composite measure of 4 tests Mean (SD): 0.142 (0.753)	NOT REPORTED	Cognitive function decline increased by 0.058 units/year after incident stroke.<br><br>Cognitive decline increased significantly after stroke relative to before stroke.<br><br>Cognitive decline increased 1.9 fold after incident stroke with cognitive function predicting mortality even after adjusting for stroke, demographic and health related factors.
Kohler 2012[10]	No reported stroke only outcomes	NOT REPORTED	Verbal fluency (estimate, p-value): intercept -0.919, 0.002 slope -0.219, 0.56;

	Verbal fluency: M=19.5, SD=5.4. (Not stroke or TIA specific)<br><br>Immediate recall: M=18.6, SD=4.0. (Not stroke or TIA specific)<br><br>Delayed recall: M=5.4, SD=2.2. (Not stroke/TIA specific)		Immediate recall (estimate, p-value): intercept -0.641, 0.003; slope -0.532, 0.09<br><br>Delayed recall (estimate, p-value): intercept -0.311, 0.01; slope -0.062, 0.68
Ghosal 2014[3]	BMSE (n = 254) - mean (SD)<br>26.48 +/- 3.4	BMSE mean (SD)<br>Year 1 (n = 197)<br>26.81 +/- 3.11<br><br>Year 2 (n = 161)<br>26.45 +/- 3.75<br><br>Year 3 (n = 141)<br>25.89 +/- 4.66	BMSE Coefficient time (standard error) over 3 years = -0.2061 (0.0937) (p = 0.028)<br><br>Cognitive dysfunction was associated with negative outcome regarding mood state affecting both basic and instrumental activities of daily living. Education was inversely related to cognitive status. Neuropsychiatric (depression and cognition), socioeconomic (lower educational level), demographic (female sex), and cultural factors can adversely affect outcome in stroke survivors.
Levine 2013[4]	3MSE: men mean =83.3 (SD 12.1), women M=85.1 (SD 11.8)<br><br>Word list delayed recall: men mean=7.0 (SD 2.9) women M=8.7 (SD 3.0)	3MSE errors increased by 22%/year in men (95% CI, 6.8% - 36.7%) and 13.2%/year in women (95% CI, 3.5% - 22.9%)<br><br>Word list improved by 0.05 words/year (95% CI -0.24 - 0.33) in men and by 0.09 words/year (95% CI -0.16 - 0.34)	Parameter (SE), p value.<br>3MSE<br>Men<br>Model A<br>post-stroke intercept: 0.60 (0.39), non-significant<br>post stroke by time interaction term: 0.17 (0.06), p<=0.01<br>post stroke change per visit: 0.20 (0.06), p<=0.01<br><br>Model B<br>post-stroke intercept: 0.29 (0.38), non-significant<br>post stroke by time interaction term: 0.16 (0.06), p<=0.01<br>post stroke change per visit: 0.17 (0.06), p<=0.01<br><br>Women<br>Model A<br>post-stroke intercept: 0.71 (0.39), p<=0.05<br>post stroke by time interaction term: 0.09 (0.04), p<=0.05<br>post stroke change per visit: 0.12 (0.04), p<=0.01<br><br>Model B

			post-stroke intercept: 0.49 (0.28), non-significant<br>post stroke by time interaction term: 0.12 (0.04), p<=0.01<br>post stroke change per visit: 0.13 (0.04), p<=0.01<br><br>Model A: included baseline age and years of education, time varying depressive symptoms (CES-D scores), time varying incident stroke, time and the incident stroke by time interaction term.<br><br>Model B added time-varying systolic BP to model A.<br><br>MMSE: 3MSE errors increased by 2.4% per year in men and increased by 3.3& per year in women. Post stroke changes in 3MSE errors were statistically significant in both men and women. Over the post stroke period 3MSE errors increased by 22% per year in men and by 13.2% per year in women.<br><br>Changes in word list scores post-stroke were not statistically significant in either sex. However, the magnitude of post-stroke change in word list scores was 1.7 times larger in women than in men.
Dik 2000[12]	Mean MMSE 26.5<br><br>Mean Immediate recall 6.8<br><br>Mean delayed recall 4.1<br><br>Mean information processing speed 20.2	No scores published but percentage that declined reported:<br>Decline in MMSE % 28.3%<br><br>Decline in Immediate recall 11.8%<br><br>Decline in delayed recall % 19.6<br><br>Decline in information processing speed % 18.8	Odds Ratio (95% CI) for Stroke patients (adjusted for age, sex, education, baseline cognition score)<br>MMSE 1.9 (1.0-3.7)<br>Immediate Recall 0.7 (0.4 – 1.6)<br>Delayed Recall 1.4 (0.7 – 2.9)<br>Information processing speed 1.2 (0.7 – 2.1)<br><br>APOE e4 carriers demonstrated a non-significantly lowered risk for MMSE decline. APOE e4 associated with declines in info processing speed and small declines for immediate and delayed recall. Of the 53 stroke patients - (n=17) had the e4 allele for APOE, (n=36) did not. Stroke patients without ApoE e4 had the lowest changes in MMSE (-1.6 points). Stroke patients with e4 showed greater declines in info processing speed (-2.0 points).
Reitz 2006[5]	NOT REPORTED	NOT REPORTED	Memory declined significantly over time (β= -1.6, p=0.005), abstract/visuospatial and language performance remained stable. A history of stroke was associated with more rapid decline in memory performance over time (β=-3.6, p=0.04). There was no relation between stroke and decline in abstract/visuospatial or language performance.<br><br>Memory and abstract/visuospatial function declined at a faster rate in men or persons who lacked the APOEe4 allele with stroke compared to women or APOEe4 carriers. This remain unchanged after adjusting for age, education, ethnic group, BMI< hypertension, heart disease, diabetes and smoking.

			The association between stroke and decline in memory performance was strongest for men and for persons without an APOE4 allele. A significant association between stroke and decline in abstract/visuospatial performance was also observed for persons without the APOE-e4 allele.
Suzuki 2013[6]	MMSE at baseline First round: median 23 (IQR: 17 - 25), Second round: median 23 (IQR 20 - 25)	MMSE (second round): third assessment (2 weeks) - Median 24 (IQR 22 - 27), fourth set of assessments median 25 (IQR 23-28)	Third set of Assessments<br>Predicted MMSE Score (logarithmic model) median 25 (IQR 22-27), model fit 0.68 (P<0.0001 for difference between actual and predicted values).<br><br>Predicted MMSE score (linear regression model): median 25 (IQR 22-28), Model fit 0.60 (P<00001 for difference between actual and predicted values (linear regression analysis)
Ben Assayag 2015[7]	Mean (SD) (Baseline)<br>All participants: MoCA 23.9 (3.3)<br>Cognitively intact at 2 years: 24.3 (3.1)<br>Cognitive Decline 2 years after stroke: 21.8 (3.6)<br><br>Mean (SD) (Baseline)<br>All participants: Computerised global cognitive score 92.5 (14.1)<br>Cognitively intact at 2 years: 93.6 (13.7)<br>Cognitive Decline 2 years after stroke: 86 (15.2)	Mean (SD) (6 months)<br>All participants: MoCA 25.3 (3.3)<br>Cognitively intact at 2 years: 25.7 (3)<br>Cognitive Decline 2 years after stroke: 22.9 (3.9)<br><br>Mean (SD) (6 months)<br>All participants: Computerised global cognitive score 94.8 (12.4)<br>Cognitively intact at 2 years: 96.1 (11.8)<br>Cognitive Decline 2 years after stroke: 87 (13.5)	Cognitive scores 6 months after stroke/TIA (23.9±3.3 versus 25.3±3.3, P<0.001 for the Montreal cognitive assessment scores; 92.5±14.1 versus 94.8±12.4, P<0.001 for the computerized global cognitive score<br><br>Univariate predictors of cognitive decline 24 months from stroke include: age greater than or equal to 75, education <12 years, white matter lesion score, Modified Rankin score 6 months after stroke, MoCA score at hospital admission, MoCA score 6 months after stroke, Berg Balance Scale 6 months after stroke (<50), the Timed Up and Go test score 6 months after stroke (>12 seconds), number of correct answers during dual-task 6 months after stroke (<15). Multivariate predictors include age greater than or equal to 75 years, TUG score (> 12secs) 6 months after stroke, MoCA score 6 months after stroke<br><br>Balance and gait are significant risk markers for cognitive status and impaired cognitive recovery after mild stroke or TIA
Tene 2016[8]	Mean (SD) (Baseline)<br>All participants: MoCA 23.8 (3.3)<br>GDS < 6: 23.8 (3.4)<br>GDS > or equals 6: 23.5 (3.2)<br><br>Mean (SD) (Baseline)<br>All participants: Computerised global cognitive score 91.8 (14.1)<br>GDS <6: 91.9 (14.6)	Mean (SD) (6 months)<br>All participants: MoCA 25.0 (3.7)<br>GDS < 6: 25.1 (3.5)<br>GDS > or equals 6: 24.2 (4.6)<br><br>Mean (SD) (6 months)<br>All participants: Computerised global cognitive score 94.1 (12.5)<br>GDS <6: 94.8 (12.1)<br>GDS > or equals 6: 89.4 (14.1)	Univariate predictors of cognitive decline 24 months from stroke include: age greater or equal to 75 years, education <12 years, ischaemic heart disease, hypertension, white matter lesion score, MoCA score at hospital admission, MoCA score 6 months after stroke Computerised global cognitive score at admission and 6 months post-stroke, GDS score at admission and 6 months posts-stoke. Multivariate predictors include MoCA at admission, age greater or equal to 75 and admission GDS score greater than or equal to 6<br><br>Depressive symptoms in poststroke/TIA patients are associated with MCI or dementia and functional deterioration at 2-year follow-up. This association occurs immediately after stroke/TIA and becomes more significant 6 months after the initial stroke.

	GDS > or equals 6: 91.3 (9.3)		
Leeds 2001[13]	CAMCOG-R<br><br>Baseline<br>TOTAL: 79.5 (median), mean 77.8 (SD 13.8)<br><br>EF: 14.0 (median), mean 13.1 (SD 4.7)<br><br>EX: median 5.0, mean 5.1 (SD 2.6)	Follow-up – significant improvement in all three mean scores:<br>TOTAL: 83.14 (SD 12.2)<br>EF 14.6 (SD 4.9)<br>EX 5.8 (SD 2.5)	Depression influenced the performance on executive function tests as well as the overall CAMCOG-R score.
Wagle 2010[14]	RBANS total scale score at baseline (median (IQR)):<br>n=104, total score baseline 73 (20).<br><br>(Cognitive impairment according to RBANS Total Index Score was defined as a score <= 77.5 points, equal to 1.5 SD below mean which is recommended cut-off score for MCI.<br><br>MMSE score at baseline (median (IQR)): n=104. MMSE=25 (7)<br><br>RBANs index score (individual tests) (median (IQR)):<br>Immediate memory 89 (25)<br>Visuospatial/constructional 82 (34)<br>Language 76 (22)	RBANS total scale score at 13 months follow up (median (IQR)):<br>n=104, total score follow up 78 (29) (p=0.001)<br><br>MMSE score at 13 months follow up (median (IQR)): n=104. MMSE=25 (9)<br><br>RBANS index score (individual tests) at 13 months (median (IQR)):<br>Immediate memory 89 (39)<br>Visuospatial/constructional 94 (45)<br>Language 82 (25)<br>Attention 65 (18)<br>Delayed memory 83 (29)<br><br>Significant differences found for visuospatial (p<0.001), delayed memory (p=0.034)<br><br>APOEe4-negative RBANS index scores at 13 months (median (IQR))<br>Immediate memory 91.0 (36.0)<br>Visuospatial/constructional 97.0 (46.0)<br>Language 84.0 (25.0)<br>Attention 70.0 (21.0)	Of the n=104, 61 (59%) were classified as cognitively impaired at baseline, compared to 52 (50%) at follow up. In total, 45 were classed as cog. Impaired at both occasions (persistent cases), 7 of the non-impaired at baseline switched to impaired at follow up, 16 were classed as cognitively impaired at baseline but switched to non-impaired at follow up (recovery cases)<br><br>A significant dose-dependent effect of the APOE-genotype in relation to overall post-stroke cognitive functioning was found at baseline and follow-up, but not pre-stroke. The e4 carriers showed a significant decline in tests related to verbal learning and memory compared to the non-carriers.<br><br>Four factors at baseline were independently associated with cognitive impairment at 13 months after acute stroke:<br><br>Previous stroke, higher IQCODE score (indicating poorer pre-stroke cognitive functioning), higher NIHSS score (indicating more severe strokes) and the presence of one or two e4-alleles.

	Attention 70 (18)<br>Delayed memory 82 (22)<br><br>APOEe4-negative RBANS index scores (median (IQR))<br>Immediate memory 94.0 (23.0)<br>Visuospatial/constructional 82.0 (33.0)<br>Language 82.0 (23.0)<br>Attention 70.0 (18.0)<br>Delayed memory 85.0 (23.0)<br>Total scale 77.0 (21.0)<br><br>APOEe4 positive RBANs index score (median (IQR))<br>Immediate memory 83.0 (25.0)<br>Visuospatial/constructional 78.0 (27.5)<br>Language 71.0 (16.5)<br>Attention 65.0 (11.5)<br>Delayed memory 76.0 (14.5)<br>Total scale 65.0 (11.5)<br><br>APOEe4-negative MMSE (median (IQR))<br>Total 25.0 (7.0)<br>Orientation for time 4.0 (2.0)<br>Orientation for place 5.0 (1.0)<br><br>APOEe4-positive MMSE (median (IQR))<br>Total 26.0 (8.0)<br>Orientation for time 4.0	Delayed memory 91.0 (29.0)<br>Total scale 83.0 (32.0)<br><br>APOEe4 positive RBANs index score (median (IQR))<br>Immediate memory 77.0 (27.0)<br>Visuospatial/constructional 85.0 (44.5)<br>Language 71.0 (25.0)<br>Attention 62.0 (8.0)<br>Delayed memory 71.0 (16.5)<br>Total scale 62.0 (17.0)<br><br>APOEe4-negative MMSE at 13 months (median (IQR))<br>Total 26.0 (8.0)<br>Orientation for time 5.0 (2.0)<br>Orientation for place 5.0 (1.0)<br><br>APOEe4-positive MMSE (median (IQR))<br>Total 22.0 (12.3)<br>Orientation for time 2.5 (4.0)<br>Orientation for place 5.0 (1.0)	

	(2.0)<br>Orientation for place 5.0 (2.0)		
Toole 2004[9]	NOT REPORTED	NOT REPORTED	Participants with a history of stroke (model 0: adjusted for 3MS) had an annualised loss of 1.6 (95% confidence interval -2.1, -1.1) points per year more than those without previous stroke.<br><br>Participants with baseline stroke (model 1: adjusted for prior 3MS, age, sex, race, education, income, smoking, hypertension, antihypertensive and antidepressant medication use, prior diabetes and prior coronary heart disease) had an average 3MS decline of 1.2 (95% confidence interval [CI]: 0.7- 1.7) points per year more than those without one.<br><br>Results demonstrate that the rate of cognitive decline after ischemic stroke is more than double that of individuals without one. In addition, a recent left hemisphere stroke causes decline 10 times as great as that experienced by persons without one and 60% more rapid than that of persons with a recent stroke in the right hemisphere.
Rowan 2007[11]	Total group<br>CAMCOG 86.6 (7.9)<br>Executive function 14.7 (4.6)<br>Language expression 16.9 (2.0)<br>Power of attention 1.8 (0.7)<br><br>Total CAMCOG at 3 months post-stroke, study group by quartile homocysteine level<br>Lowest 86.6 (8.3)<br>2nd 86.5 (8.1)<br>3rd 87.8 (8.3)<br>Highest 85.7 (6.6)<br><br>Executive function at 3 months post-stroke, study group by quartile homocysteine level<br>Lowest 15.1 (15.1)	Total group (mean changes (SD) between 3 and 27 months post stroke)<br>CAMCOG -0.6 (7.8)<br>Executive function -2.1 (4.7)<br>Language expression -0.4 (1.8)<br>Power of attention 0.1 (0.4)<br><br>Total CAMCOG (mean changes (SD) between 3 and 27 months post stroke)<br>Lowest -2.3 (8.4)<br>2nd 1.1 (8.4)<br>3rd -0.2 (6.8)<br>Highest -1.7 (7.4)<br><br>Executive function (mean changes (SD) between 3 and 27 months post stroke)<br>Lowest -2.6 (4.3)<br>2nd -2.3 (5.9)<br>3rd -1.5 (3.6)<br>Highest -1.7 (4.5)<br><br>Language expression (mean changes (SD) between 3 and 27 months post stroke)	51% of elderly non-demented stroke patients have hyperhomocysteinaemia at 3 months post stroke. 79% of elderly stroke patients scored above 80 points on the CAMCOG at 27 months post stroke.<br><br>Found no associations between homocysteine levels and cognitive change, therefore 3 month post-stroke homocysteine measurement may not predict cognitive decline

	2nd 14.1 (5.3)<br>3rd 15.0 (4.4)<br>Highest 14.5 (3.6)<br><br>Language expression at 3 months post-stroke, study group by quartile homocysteine level<br>Lowest 17.1 (2.0)<br>2nd 16.9 (2.2)<br>3rd 17.2 (1.6)<br>Highest 16.6 (2.0)<br><br>Power of Attention at 3 months post-stroke, study group by quartile homocysteine level<br>Lowest 1.7 (0.5)<br>2nd 1.8 (0.7)<br>3rd 1.7 (0.5)<br>Highest 1.9 (0.9)	Lowest -0.5 (1.7)<br>2nd -0.3 (1.9)<br>3rd -0.2 (2.0)<br>Highest -0.5 (1.4)<br><br>Power of Attention (mean changes (SD) between 3 and 27 months post stroke)<br>Lowest 0.1 (0.4)<br>2nd 0.1 (0.6)<br>3rd 0.1 (0.4)<br>Highest 0.0 (0.3)	

Abbreviations

3MS, modified mini mental; 3MSE, modified mini mental examination; ACS, acute coronary syndrome; ACT, Alphabet Coding Task; ADL, activities of daily living; aMCI, amnestic mild cognitive impairment; AVLT, Auditory Verbal Learning Test; BMI, body mass index; BMSE, Bengali versions of the Mini Mental State Examination; CAMCOG-R, Cambridge Cognitive Assessment; Cambridge Cognitive Assessment (Revised); CES-D, Center for Epidemiologic Study-Depression; CI, Confidence interval; EF, Total executive functioning subtests of the CAMCOG-R (fluency + similarities+ ideational fluency + visual reasoning); CERAD, Consortium to Establish a Register for Alzheimer’s Disease; EX, New executive functioning tests (ideational fluency + visual reasoning); FIM, functional independence measure; GDS, Geriatric Depression Score; IQCODE, Informant questionnaire on Cognitive Decline in the Elderly; IQR, interquartile range; LACS, Lacunar stroke; MCI, mild cognitive impairment; MMSE, Mini Mental State Examination; MoCA, Montreal Cognitive Assessment; OCSP, The Oxfordshire Community Stroke Project; OR, odds ratio; PACS, Partial anterior circulation stroke; POCS, Posterior circulation stroke; Repeatable Battery for the Assessment of Neuropsychological Stats (RBANS); RCPM, Raven’s Colored Progressive Matrices; SD, Standard Deviation; SE, standard error; SFE, social functioning exam; TACS, Total anterior circulation stroke; TIA, transient ischaemic attack; TUG, Timed Up and Go

Supplemental References:

1. Comijs HC, Kriegsman DM, Dik MG, Deeg DJ, Jonker C, Stalman WA. Somatic chronic diseases and 6-year change in cognitive functioning among older persons. *Arch Gerontol Geriatr*. 2009;48:191-196.

2. Rajan KB, Aggarwal NT, Wilson RS, Everson-Rose SA, Evans DA. Association of cognitive functioning, incident stroke, and mortality in older adults. *Stroke*. 2014;45:2563-2567.

3. Ghosal MK, Burman P, Singh V, Das S, Paul N, Ray BK, Hazra A, Banerjee TK, Basu A, Chaudhuri A, Das SK. Correlates of functional outcome among stroke survivors in a developing country - a prospective community-based study from india. *J Stroke Cerebrovasc Dis*. 2014;23:2614-2621.

4. Levine DA, Haan MN, Langa KM, Morgenstern LB, Neuhaus J, Lee A, Lisabeth LD. Impact of gender and blood pressure on poststroke cognitive decline among older latinos. *J Stroke Cerebrovasc Dis.* 2013;22:1038-1045.

5. Reitz C, Luchsinger JA, Tang MX, Manly J, Mayeux R. Stroke and memory performance in elderly persons without dementia. *Arch Neurol*. 2006;63:571-576.

6. Suzuki M, Sugimura Y, Yamada S, Omori Y, Miyamoto M, Yamamoto J. Predicting recovery of cognitive function soon after stroke: Differential modeling of logarithmic and linear regression. *PloS one*. 2013;8:e53488.

7. Ben Assayag E, Shenhar-Tsarfaty S, Korczyn AD, Kliper E, Hallevi H, Shopin L, Auriel E, Giladi N, Mike A, Halevy A, Weiss A, Mirelman A, Bornstein NM, Hausdorff JM. Gait measures as predictors of poststroke cognitive function: Evidence from the tabasco study. *Stroke*. 2015;46:1077-1083.

8. Tene O, Shenhar-Tsarfaty S, Korczyn AD, Kliper E, Hallevi H, Shopin L, Auriel E, Mike A, Bornstein NM, Assayag EB. Depressive symptoms following stroke and transient ischemic attack: Is it time for a more intensive treatment approach? Results from the tabasco cohort study. *J Clin Psychiatry*. 2016;77:673-680.

9. Toole JF, Bhadelia R, Williamson JD, Veltkamp R. Progressive cognitive impairment after stroke. *J Stroke Cerebrovasc Dis.* 2004;13:99-103.

10. Kohler M, Kliegel M, Kaduszkiewicz H, Bachmann C, Wiese B, Bickel H, Mösch E, Weyerer S, Werle J, Fuchs A, Pentzek M, Leicht H, König HH, Luppa M, Riedel-Heller S, Jessen F, Maier W, Scherer M, Wagner M; Ageing, Cognition and Dementia in Primary Care Patients (AgeCoDe) study group. Effect of cardiovascular and metabolic disease on cognitive test performance and cognitive change in older adults. *J Am Geriatr Soc*. 2012;60:1286-1291.

11. Rowan EN, Dickinson HO, Stephens S, Ballard C, Kalaria R, Kenny RA. Homocysteine and post-stroke cognitive decline. *Age Ageing*. 2007;36:339-343.

12. Dik MG, Deeg DJ, Bouter LM, Corder EH, Kok A, Jonker C. Stroke and apolipoprotein e epsilon4 are independent risk factors for cognitive decline: A population-based study. *Stroke*. 2000;31:2431-2436.

13. Leeds L, Meara RJ, Woods R, Hobson JP. A comparison of the new executive functioning domains of the camcog-r with existing tests of executive function in elderly stroke survivors. *Age Ageing*. 2001;30:251-254.

14. Wagle J, Farner L, Flekkoy K, Wyller TB, Sandvik L, Eiklid KL, Fure B, Stensrød B, Engedal K. Cognitive impairment and the role of the apoe epsilon4-allele after stroke--a 13 months follow-up study. *Int J Geriatr Psychiatry.* 2010;25:833-842.
